# Cancer burden among HIV-positive persons in Nigeria: preliminary findings from the Nigerian AIDS-cancer match study

**DOI:** 10.1186/1750-9378-9-1

**Published:** 2014-03-05

**Authors:** Sally N Akarolo-Anthony, Luigino Dal Maso, Festus Igbinoba, Sam M Mbulaiteye, Clement A Adebamowo

**Affiliations:** 1 Department of Nutrition, Harvard School of Public Health, 677 Huntington Avenue, Boston, MA, USA; 2 Office of Strategic Information and Research, Institute of Human Virology, Abuja, Nigeria; 3 Epidemiology and Biostatistics Unit, Aviano Cancer Center, IRCCS, Aviano, Italy; 4 Abuja Cancer Registry, National Hospital Abuja, Abuja, Nigeria; 5 Division of Cancer Epidemiology and Genetics, National Cancer Institute, Bethesda, MD, USA; 6 Institute of Human Virology and Greenebaum Cancer Center, University of Maryland School of Medicine, Baltimore, MD, USA

**Keywords:** HIV, Cancer, AIDS-cancer match, Registry linkage, Nigeria

## Abstract

**Background:**

Although Nigeria has a large HIV epidemic, the impact of HIV on cancer in Nigerians is unknown.

**Methods:**

We conducted a registry linkage study using a probabilistic matching algorithm among a cohort of HIV positive persons registered at health facilities where the Institute of Human Virology Nigeria (IHVN) provides HIV prevention and treatment services. Their data was linked to data from 2009 to 2012 in the Abuja Cancer Registry. Match compatible files with first name, last name, sex, date of birth and unique HIV cohort identification numbers were provided by each registry and used for the linkage analysis. We describe demographic characteristics of the HIV clients and compute Standardized Incidence Ratios (SIRs) to evaluate the association of various cancers with HIV infection.

**Results:**

Between 2005 and 2012, 17,826 persons living with HIV (PLWA) were registered at IHVN. Their median age (Interquartile range (IQR)) was 33 (27–40) years; 41% (7246/17826) were men and 59% (10580/17826) were women. From 2009 to 2012, 2,029 clients with invasive cancers were registered at the Abuja Cancer Registry. The median age (IQR) of the cancer clients was 45 (35–68) years. Among PLWA, 39 cancer cases were identified, 69% (27/39) were incident cancers and 31% (12/39) were prevalent cancers. The SIR (95% CI) for the AIDS Defining Cancers were 5.7 (4.1, 7.2) and 2.0 (0.4, 3.5), for Kaposi Sarcoma and Cervical Cancer respectively.

**Conclusion:**

The risk of Kaposi Sarcoma but not Cervical Cancer or Non-Hodgkin’s Lymphoma, was significantly increased among HIV positive persons, compared to the general population in Nigeria.

## Background

HIV infection is associated with an increased risk of diverse cancers. Classically, the Centers for Disease Control and Prevention (CDC) designated Kaposi sarcoma (KS) cervical cancer (CC) and non-Hodgkin’s lymphoma (NHL) as AIDS defining cancers (ADCs) because of the association of these cancers with increased risk of HIV infection. The advent of highly active antiretroviral therapy (HAART) in 1996 has led to significant reduction in the incidence and prevalence of these ADCs [1]. Nevertheless, the risk of ADCs remains high in many low and middle income countries (LMIC) where there are significant populations with HIV infection who are not on treatment. In contrast, in countries with mature HIV epidemics and combination ART, there is a modest increase in other cancers which have been designated non-AIDS defining cancers (NADCs), including anal, head and neck, lung and liver cancers, Hodgkin’s lymphoma, and melanoma [2-6].

Most of the studies conducted to investigate the relationship between HIV infection and malignancies in sub-Saharan Africa to date have largely emanated from East and Southern Africa and this may not accurately reflect the pattern of AIDS associated malignancies in Africa [7-13]. Furthermore only one of these African studies was population based and it showed that about 70% of cancers occurring in the studied HIV infected population in Uganda were ADCs. The risks of five other NADCs - cancers of the kidney, thyroid, uterus, conjunctiva and Hodgkin’s lymphoma - were also increased [12]. Apart from such systematic studies, other reports from Africa were based on hospital or pathology case series with a majority of such studies emanating from Nigeria, West Africa. Table 1 is a summary of the AIDS-associated malignancy studies among adults in Africa from January 2001 to December 2012.

**Table 1:** Summary of AIDS-associated malignancy studies among adults in Africa, January 2001 – December 2012

**Reference**	**Study type**	**Study population**	**Study period**	**Key findings**
Ocheni (2004) [14]	Case series	University College Hospital, Ibadan, Nigeria	Oct 2001 – June 2002	KS prevalence = 0.01%
		n = 6; age range =29-35; m:f = 1:1.4		
				CC prevalence = 0.01%
				NHL prevalence = 0.02%
Cisse (2007) [15]	Case series	Hôpital du Point G, Bamako, Mali	Oct 2004 – Sept 2005	KS prevalence = 1.6%
		n = 37; mean age = 39.5; m:f = 1:1		
Ibekwe (2011) [16]	Case series	University college hospital Ibadan, Nigeria	Oct 2008 - Sep 2009	KS prevalence =0.5%
		n = 13; age range = 26 – 56; m:f = 1:1		
Abdus-Salam (2006) [17]	Case series	University college hospital Ibadan, Nigeria	2005- 2007	CC prevalence = 2.7%
		n = 6		
Bolarinwa (2009) [18]	Case series	Obafemi Awolowo university teaching hospital, Ile-Ife, Nigeria	Jan 1993 – Aug 2008	NHL = 1.5%
		n = 9; age range = 24 – 60; m:f = 1:1		
Chu (2010) [19]	Case series	3 primary care HIV clinics, Khayelitsha, Cape town, South Africa	May 2001 – Jan 2007	KS prevalence = 2.4%
		n = 189; age IQR = 29 – 41; m:f = 1.4:1		
Iregbu (2006) [20]	Case series	National hospital Abuja, Nigeria	Jan 2002 – July 2005	KS prevalence = 0.8%
		n =15; age = 20–49; m:f = 2:1		
Sitas (2000) [13]	Case–control	Chris Hani-Baragwanath, Hillbrow, New Johannesburg Hospitals, South Africa	1995 – 1999	KS* = 21.9 (12.5, 38.6)
		n CC = 167; n KS = 89; n = NHL = 23		NHL* = 5.0 (2.7-9.5)
				CC* = 1.6 (1.1-2.3)
Stein (2008) [21]	Case–control	Chris Hani Baragwanath, Hillbrow & New Johannesburg Hospitals, Johannesburg, South Africa	Mar 1995–June 2004	Age group 18 – 34 only
		n CC = 167; n KS = 89; n NHL = 50		KS* = 58.6 (27.6, 124.5)
		Age range = 18 – 55^+^		NH* = 6.6 (3.7, 12.0)
				CC* = 1.5 (0.9, 2.4)
Mbulaiteye (2006) [12]	AIDS cancer registry match	Kampala cancer registry and AIDS support organization, Kyadondo, Uganda	Oct 1998 – Dec 2002	KS^#^ = 6.4 (4.8-8.4)
		Number of incident cancers = 137; age IQR = 25-35		NHL^#^ = 6.7 (1.8-17)
				CC^#^ = 2.4 (1.1-1.4)
Chaabna (2011) [22]	Population-based cancer registries	Kyadondo, Uganda; Harare, Zimbabwe; Setif, Algeria; Sousse, Tunisia; and Gharbiah Egypt	1998 - 2002	KS^P^ = <0.3– 26.3/100,000
				CC^P^ = <8-34.8/100,000
				NHL^P^ = 2.4 – 11.9/100,000

n = number of cases with HIV; m: f = male: female; *OR and 95% confidence interval; ^#^SIR and 95% confidence interval; ^P^Incidence.

Nigeria being the most populous country in Africa, with an estimated population of 170,123,740 (41% of whom are below age 15 years) in 2012 [23] and the third highest population of persons living with HIV/AIDS (PLWA) in the world, is expected to bear a significant proportion of the projected AIDS associated cancer burden in Africa. The incidence and prevalence of cancer among PLWA in Nigeria is not known. This lack of data hampers the ability to formulate sound policy for clinical and public health intervention. The successful implementation of record-linkage studies in different parts of the world, e.g. Uganda, India, suggests that this may be a feasible approach to the study of AIDS associated cancers in Nigeria.

We conducted this study to evaluate the spectrum of cancers diagnosed among PLWA in Nigeria using the record-linkage technique.

## Results

**Figure 1:**
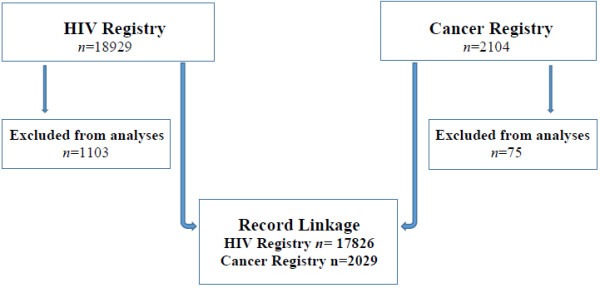
Participants flow chart.

We excluded 1103 (6%) and 75 (4%) clients from the HIV and cancer registries respectively, because they had missing data on age, date of diagnosis or several demographic variables. The participant flow is summarized in Figure 1 and the characteristics of the population study are summarized in Table 2. Between 2005 and 2012, 17,826 clients were registered by the HIV registry. The median age (Interquartile range (IQR)) of the HIV clients was 33 (27–40) years. On average, ~2000 clients were registered with the HIV registry per year, of whom 41% (7,246/17,826) were men and 59% (10,580/17,826) were women. From 2009 to 2012, 2,029 clients with invasive cancers were registered at the cancer registry. The median age (IQR) of the cancer clients was 45 (35–68) years. On average, ~500 clients were registered with the cancer registry/year, 36% (728/2,029) of these were men and 64% (1,301/2,029) were women.

**Table 2:** Baseline characteristics of persons registered with the Institute of Human Virology Nigeria (IHVN) HIV registry

**Characteristics**	**HIV registry (N = 17,826)**
Sex, n (%)	
Male	7,246 (41)
Female	10,580 (59)
Age (years), n (%)	
0 – 14	1,389 (8)
15 – 24	1,237 (7)
25 – 34	6,980 (39)
35 – 44	5,567 (31)
45 – 54	2,091 (12)
55 – 64	476 (2.5)
65+	86 (0.5)
Median age at diagnosis, IQR (years)	33 (27–40)
Calendar year of registration, n (%)	
2005	3,336 (18)
2006	4,409 (25)
2007	3,017 (17)
2008	2,414 (14)
2009	2,025 (11)
2010	1,134 (6)
2011	833 (5)
2012	658 (4)

Among PLWA, 39 cancer cases were identified, 31% (12/39) were prevalent cancers and 69% (27/39) were incident cancers. All the prevalent cancers occurred from 34 months before to 2 months after registration, while 44% (12/27) and 56% (15/27) of the incident cancers occurred in the early and late incident periods respectively. Also, 26% (10/39) of the cancers identified were among men and 74% (29/39) among women. The ADCs identified were KS and CC; while the NADCs were breast, ovary, prostate, liver, anal, eye and other non-epithelial skin cancers. The ADCs and NADCs accounted for 41% (16/39) and 59% (23/39) of the cancers among PLWA respectively. The SIR (95% CI) for the ADCs were 5.7 (4.1, 7.2) and 2.0 (0.4, 3.5), for KS and CC respectively. We observed 9 cases of breast cancer, but the SIR was not significantly increased (SIR 1.6, 95% CI 0.2, 3.0). Table 3 shows the types, ICD and morphology codes of the observed cancers among HIV-infected persons.

**Table 3:** Spectrum of observed cancers and standardized incidence ratios for HIV-infected persons

**Cancer type**	**ICD code**	**Morphology code**	**N (%)**	**Incidence/100,000 person years**	
				**Crude rate**	**SIR (95% CI)**
**AIDS defining cancers**					
Kaposi sarcoma	C46	9140	8 (21)	4.9	5.7 (4.1, 7.2)
Cervical cancer	C53	8070	8 (21)	7.8	2.0 (0.4, 3.5)
**Non AIDS defining cancers**					
Anus	C21	8010	1 (2)	0.6	0.3 (0, 17.8)
Liver	C22	8170, 8174	3 (8)	1.8	0.5 (0, 5.1)
Other non-epithelial skin	C44	8070	3 (8)	1.8	4.0 (0, 8.5)
Breast	C50	8010, 8521	9 (23)	8.8	1.6 (0.2, 3.0)
Ovary	C56	8010, 8310	2 (5)	2.0	3.6 (0, 20.1)
Prostate	C61	8010	1 (2)	0.6	0.4 (0, 16.9)
Eye	C69	8052	1 (2)	0.6	1.5 (0, 18.1)
Other, non-specified		8001	3 (8)	1.8	0.5 (0, 4.7)

## Discussion

This study is the first to use record linkage analysis to estimate cancer incidence among PLWA in West Africa. Among the ADCs, only KS was significantly increased among HIV positive persons and there was no statistically significant increase in incidence of NHL, CC and NADCs. These results for KS and CC are consistent with the findings from studies conducted in other countries where increased risk of KS among HIV positive persons and no association between CC and HIV was found [24-26]. Although several studies suggest that the burden of NHL is increased among HIV positive persons in Africa [12,13,27,28], studies from West Africa have not supported this, just like our results. Similar to Bolarinwa *et al.*, we identified several cases of NHL in the general population but none of these were matched in the HIV registry [18].

The relatively low incidence of cancers among HIV positive persons in Nigeria is probably multifactorial. An important possibility may be due to lack of cancer diagnosis. Given the size of the HIV/AIDS epidemic and main focus to diagnose and treat HIV or roll out prevention programs, care providers may miss clinical features suggestive of cancer in persons with HIV infection or fail to investigate those clients thoroughly. Although not well documented, our causal impression is that some physicians who are concerned about occupational exposure to HIV may provide limited quality services to HIV positive persons [29,30]. Finally, the infrastructure to diagnose, refer, and register patients may also have caused low levels of utilization of diagnostic procedures needed to confirm cancer diagnosis in PLWA [29].

There is some evidence that ascertainment of cancer by the ABCR may not be complete. For example, the Kampala Cancer Registry, which covers a population of about 2.1 million, registered 4235 cancer cases during 2007 - 2009 [31]. ABCR, which covers a population of about 1.4 million, registered 1815 cancer cases from 2009–2011 registered. This may be due to a longer experience with cancer registration resulting in more complete data or a lifestyle with a higher cancer risk profile in Uganda. We also note that the HIV prevalence is higher in Kyadondo compared to Abuja, 15% and 8.6%, respectively [12,32], which may lead to a higher burden of HIV-associated cancers, such as KS in Uganda. In this regard, it is worth noting that the prevalence of human herpesvirus 8 is lower in Nigeria compared to Uganda [33], which would also explain the lower prevalence of cancer in ABCR, particularly KS. Our study highlights the opportunity to improve case finding methods of cancer registries in Nigeria. Similar to other countries in Africa, the infrastructure for population-based cancer registries is poorly developed and not fully linked to the HIV screening and registration process. Increased utilization of electronic medical records and databases, improved collaboration between registries and a multidisciplinary approach to data collection and management will improve case ascertainment of HIV and cancer cases, their report to registries and the linkage of registry data. A separate study evaluating the completeness of data obtained by the ABCR is under way.

Other possible explanations for the low cancer incidence include the availability of HAART, which would be associated with improved immune function and reduced risk of AIDS-associated cancers, such as KS [34,35]. The coverage of HAART in HIV positive persons in Nigeria is relatively low (24% of individuals who should be on HAART [36]), thus the contribution of this factor is uncertain. Loss to follow-up is another possible explanation [36], as is competing mortality from common diseases, such as tuberculosis and malaria.

Our study is also limited by the small sample size thus we were unable to compare prevalent versus incident cancers and we lacked sufficient power to detect small differences that may exist between the general population and the HIV positive population. Another limitation is lack of unique individual personal identification numbers, such as social security numbers, which would increase the specificity and accuracy of the match. Nevertheless, thorough clerical review of the linked records increased our confidence in the matched records in study. Additional research is needed to understand and improve the specificity of matches in resource-limited settings, where unique individual identification numbers are generally lacking. As the HIV positive population in Nigeria continues to age, coverage with HAART improves and the registry systems are strengthened, more record linkage studies to examine the growing public health impact of cancers occurring in people living with HIV/AIDS will be warranted.

## Conclusions

We used record-linkage analysis to evaluate AIDS-defining cancers in Nigeria and provide insights about their risk. Consistent with the findings from other studies, the risk of Kaposi Sarcoma but not Cervical Cancer or Non-Hodgkin’s Lymphoma, was significantly increased among HIV positive persons, compared to the general population in Sub-Saharan Africa. Further studies are required to more thoroughly examine the relationship between cancers and HIV in West Africa and its determinants.

## Methods

### Study population

Abuja where this study was conducted is Nigeria’s Federal Capital Territory, it had a population of 1.4 million in 2006 [37]. Because of its role as the capital of Nigeria, individuals from all the country’s ethnic groups, tribes and religions live there. Muslims make up approximately 50% of the population, Christians 40%, while the remainder adhere to indigenous beliefs. Abuja has five districts and several surrounding towns and villages. The estimated HIV prevalence in Abuja FCT was 8.6% in 2010, the fifth highest in Nigeria [32].

### HIV registry

The study population is a cohort of 18,929 HIV positive persons, who were registered at the Institute of Human Virology Nigeria (IHVN) HIV Care and Treatment programs in Abuja, Nigeria between 2005 and 2012. The IHVN HIV cohort was established in 2005, and has accrued ~2,000 new clients per year on average, of whom the majority (>90%) have Abuja as their permanent address. Upon registration, each person is given a unique HIV cohort identification number, in addition to the hospital number. Information on age, date of birth, first name and last name, sex, marital status, educational level, occupation, address (street/city/state) and phone number are obtained. In addition, anthropometric measurements and physical examinations are performed, biologic samples are collected for quantitative and qualitative tests and referrals to appropriate departments are made when indicated. More than 90% of the clients have complete demographic information.

## Cancer registry

Abuja Cancer Registry (ABCR) is a population based cancer registry established in 2009. It covers the population of Abuja where majority of its clients (>97%) permanently reside. National Hospital Abuja (NHA), which is located in one of Abuja’s main districts, and University of Abuja Teaching Hospital (UATH), which is located in the main suburban district of Abuja are the main sources of data for the ABCR. Other ABCR data sources include public and private hospitals, and free-standing pathology laboratories in Abuja. Cancer registrars visit the data sources once a month to perform medical chart abstraction and identify new cases of cancer diagnosed during the month. For each new case, hospital number, first and last names, age and date birth, sex, tribe, address, date of diagnosis, tumor site and histology are collected.

### Registry linkage

The registry linkage was performed with a probabilistic matching algorithm, using the software for automated linkage in Italy (SALI) software. SALI is a software designed for a linkage analysis of small registries, with few expected linked cases [38]. Files with first name, last name, sex, date of birth and HIV or cancer registration numbers were provided by each registry and used for the linkage analysis. The names were edited by deleting titles, prefixes, suffixes and by correcting common variations in spelling in order to improve match sensitivity. The registry linkage was conducted in collaboration with the developers of SALI. Linkage was done by researchers who were independent of the HIV and cancer registries. To ascertain the validity of the linkage analysis, an extensive review of the medical records was conducted, to confirm the identifiers and diagnosis on all matched cases.

### Statistical analysis

Descriptive analyses were performed to characterize the demographic attributes of the clients in the IHVN and ABCR registries. Cancer incidence rates among HIV positive persons, were estimated by dividing the number of cancers by person-years of follow-up. In addition, we categorized cancers in persons with HIV or AIDS occurring 60 months before to 3 months after registration at IHVN’s HIV treatment and prevention sites as prevalent; cancers occurring 4 to 27 months after registration as early-incident; and cancers occurring 28 months after registration as late-incident cancers [12]. Standardized incidence ratios (SIRs), a ratio of the number of cancers observed among subjects, to the number of cancers expected, were estimated and used to evaluate the association of various cancers with HIV infection in the incident period. The number of expected cases for each cancer type was calculated as a sum of the product of person-time of HIV positive clients and the corresponding sex, age and period specific cancer rates from the ABCR. The source of the population count was Nigeria’s National Population Council (NPC) data, which is stratified by age, sex and geographic location [37]. As in the Ugandan record-linkage study, we considered cancers to be associated with HIV infection if SIRs were significantly increased in the incident period [12]. Statistical analyses were performed with SAS for UNIX statistical software (version 9.2; SAS Institute).

## Consent

Written informed consent was obtained from the patient for the publication of this report and any accompanying images.

## Ethics

The study was conducted according to the Nigerian National Code for Health Research Ethics and the Declaration of Helsinki. Ethical approval to conduct this study was obtained from the Institute of Human Virology Health Research Ethics Committee.

## Abbreviations

ABCR: Abuja cancer registry; ADC: AIDS defining cancers; AIDS: Acquired immunodeficiency syndrome; CC: Cervical cancer; CDC: Center for disease control; HAART: Highly active antiretroviral therapy; HIV: Human immunodeficiency virus; KS: Kaposi sarcoma; ICD: International classification of diseases; IHVN: Institute of human virology Nigeria; NACA: National agency for the control of AIDS; NADC: Non-AIDS defining cancers; NHL: Non-hodgkin’s lymphoma; PLWA: People living with HIV/AIDS; SALI: Software for automated linkage in Italy.

## Competing interests

The authors declare that they have no competing interest.

## Authors’ contributions

SNA prepared the linkage files, performed the statistical analyses and drafted the manuscript. LDM conducted the registry linkage. FI contributed to the revision of the manuscript. SM coordinated the study and provided critical revisions of the manuscript. CAA conceived the study, obtained funds, contributed to the study design of the study and provided critical revisions of the manuscript. All authors read and approved the final manuscript.
